# Phosphorylation of distal C-terminal residues promotes TRPV4 channel activation in response to arachidonic acid

**DOI:** 10.1016/j.jbc.2025.108260

**Published:** 2025-02-03

**Authors:** Aravind Parthasarathy, Andriy Anishkin, Yangjing Xie, Kostiantyn Drachuk, Yoshinori Nishijma, Juan Fang, Sevasti B. Koukouritaki, David A. Wilcox, David X. Zhang

**Affiliations:** 1 Department of Medicine, Cardiovascular Center, Medical College of Wisconsin, Milwaukee, Wisconsin, USA; 2 Department of Biology, University of Maryland, College Park, Maryland, USA; 3 Department of Pediatrics, Medical College of Wisconsin, Milwaukee, Wisconsin, USA; 4 Children's Research Institute, Children's Wisconsin, Milwaukee, Wisconsin, USA; 5 Department of Pharmacology and Toxicology, Medical College of Wisconsin, Milwaukee, Wisconsin, USA

**Keywords:** transient receptor potential vanilloid 4 channels (TRPV4 channels), arachidonic acid (AA), protein kinase C (PKC), protein kinase A (PKA), protein phosphorylation, homology modeling

## Abstract

Transient receptor potential vanilloid 4 (TRPV4) is a Ca^2+^-permeable channel activated by diverse physical and chemical stimuli, including mechanical stress and endogenous lipid arachidonic acid (AA) and its metabolites. Phosphorylation of TRPV4 by protein kinase A (PKA) and protein kinase C (PKC) is a predominant mechanism for channel regulation, especially in the cytoplasmic domains due to their importance in protein assembly, and channelopathies. However, studies corresponding to phosphorylation sites for these kinases remain incomplete. We investigated the role of Ser-823 residue as a potential phosphorylation site in regulating TRPV4 activity and chemical agonist-induced channel activation. Using mass spectrometry, we identified a new phosphorylation site Ser-823 residue and confirmed the previously known phosphorylation site Ser-824 in the C-terminal tail. The low level of phosphorylation at Ser-823 was stimulated by PKC and to a lesser extent by PKA in human coronary artery endothelial cells (HCAECs) and human embryonic kidney 293 (HEK 293) cells. AA-induced TRPV4 activation was enhanced in the phosphomimetic S823E but was blunted in the S823A/S824A mutants, whereas the channel activation by the synthetic agonist GSK1016790A was unaffected. Further, TRPV4 activation by AA but not GSK1016790A was blunted or abolished by PKA inhibitor alone or in combination with PKC inhibitor, respectively. Using computational modeling, we refined a previously proposed structural model for TRPV4 regulation by Ser-823 and Ser-824 phosphorylation. Together, these results provide insight into how stimuli-specific TRPV4 activation is regulated by the phosphorylation of discrete residues (*e.g.*, Ser-823 and Ser-824) in the C-terminal domains of the TRPV4 channel.

TRPV4, the fourth member of the transient receptor potential (TRP) vanilloid subfamily, is a Ca^2+^-permeable cation channel implicated in a broad range of physiological processes from regulation of vascular tone, angiogenesis, osmoregulation, bone homeostasis, inflammation, to nociception (1, 2, 3, 4, 5, 6). In mammals, TRPV4 channels are expressed in vascular tissues (endothelial and smooth muscle cells), neurons, pancreatic cells, skin, eyes, bone, and muscular and urinary systems (7, 8, 9, 10, 11, 12). As a multifunctional ion channel, TRPV4 can be activated or sensitized by diverse physical and chemical stimuli including hypotonic cell swelling (13), shear stress (14, 15), warm temperature (16), low pH (17), synthetic agonists such as phorbol esters (18) and GSK1016790A (19), and endogenous lipid mediators such as phosphatidylinositol 4,5- bisphosphate (PIP_2_) (20), endocannabinoids (21), arachidonic acid (AA), and its metabolites (22, 23, 24).

Remarkably, over 80 genetic mutations in TRPV4 have been identified in various channelopathies including ∼20 skeletal and peripheral nervous system disorders in humans (25, 26, 27). TRPV4 dysfunction also contributes to many acquired pathological conditions in the vasculature, heart, kidney, and other systems (28, 29, 30, 31). The precise mechanisms of TRPV4-related channelopathies remain poorly understood; however, in many disorders caused by TRPV4 mutations or non-genetic factors, an increased Ca^2+^ influx through the channel results in intracellular Ca^2+^ overload and subsequent cell degeneration or apoptosis (32). It is of note that TRPV4, being constitutively active in cells, has a relatively large single-channel conductance (∼30–60 pS at −60 mV) and is also more permeable to Ca^2+^ than other cations (P_Ca_/P_Na_ = 6–10) (33, 34). By comparison, the L-type voltage-gated Ca^2+^ channel has a single-channel conductance of 3.5 pS or 10-fold lower than that of TRPV4 (35).

Similar to other TRP vanilloid (TRPV) channels, TRPV4 is a tetramer assembled from four identical subunits and shares an overall conserved architecture with voltage-gated K^+^ (Kv) channels in the transmembrane domain (TMD), consisting of a voltage-sensor-like domain (S1-S4), an S4-S5 linker, and a pore domain (S5, P-loop, and S6), followed by the signature TRP helix (34, 36). This TRP helix is a unique feature found in most members of TRP channels: it runs under the S1-S4 transmembrane helices of the same subunit and lays almost parallel to the inner surface of the bilayer membrane. The intracellular “skirt” of TRPV4 contains an N-terminal ankyrin repeat domain (ARD), a three-stranded β-sheet linker domain, and a more distal C-terminal tail that is only partially resolved in recent cryo-electron microscopy (cryo-EM) structures of full-length human TRPV4 (37, 38). Although not fully revealed by cryo-EM like other TRP channels (39), the C-terminus of TRPV4 plays an important role in regulating TRPV4 channel activity, protein assembly, and plasma membrane trafficking (40, 41). The C-terminal tail also harbors a putative autoinhibitory domain (AID) that maintains the closed state of TRPV4 likely through electrostatic interactions, and disruption of these interactions leads to a gain of function (GOF) of TRPV4 and channelopathies (42). Therefore, a better understanding of TRPV4 cytoplasmic domains can provide insights into the fundamental pathophysiology of this channel.

Protein kinases are highly dynamic molecular switches involved in many biological processes including the regulation of ion channel activity (43, 44, 45). In addition to stimulus-dependent activation, TRPV channels such as TRPV4 and the closely related TRPV1 are tightly regulated by protein phosphorylation through different tyrosine and serine/threonine kinases such as protein kinase A (PKA) and protein kinase C (PKC) (46, 47, 48, 49). For example, TRPV4 phosphorylation in the cytoplasmic domains (Ser-162, Thr-175, and Ser-189 residues by PKC and Ser-824 residue by PKA) enhances TRPV4 activation induced by hypotonic cell swelling or other stimuli (50, 51, 52, 53, 54). Our previous studies have further established an important role of Ser-824 phosphorylation in TRPV4 activation by AA in HCAECs (human coronary artery endothelial cells) and HCAs (human coronary arterioles) (23, 54). While Ser-824 residue phosphorylation by PKA has been well established, the neighboring Ser-823 residue (a potential phosphorylation candidate) in the C-terminus of the channel has been less explored for its role in TRPV4 channel activity/regulation. Interestingly, AA-induced TRPV4 activation in HCAECs was only blunted by S824A mutation but almost abolished by PKA inhibition, suggesting that additional phosphorylation sites may be involved (54). In this study, we report that PKC phosphorylates Ser-823 in HCAECs, and HEK 293 cells and this phosphorylation enhances TRPV4 activation by AA. We further refine a previously proposed structural model to provide insight into the potential mechanism of Ser-823/Ser-824 phosphorylation in the sensitization of this ion channel.

## Results

### Ser-823 residue of TRPV4 is phosphorylated mainly through the PKC pathway in HCAECs and HEK 293 cells

**Figure 1:**
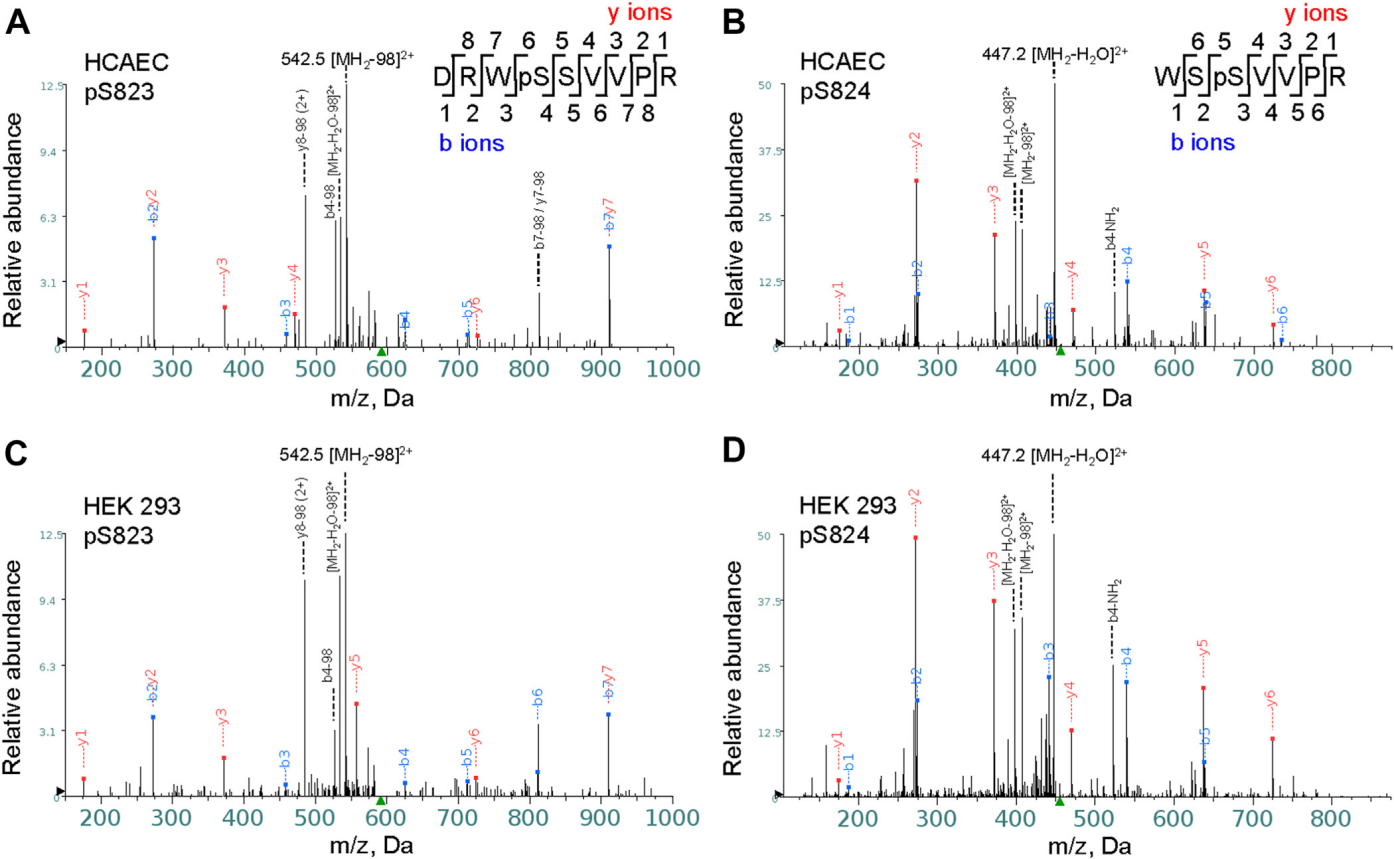
**LC-MS/MS detection of TRPV4 phosphorylation at Ser-823 and Ser-824 residues.** HCAECs were transduced with plasmids encoding human TRPV4-GFP in a lentiviral vector for 96 h, and HEK 293 cells were transfected with TRPV4-GFP plasmids for 48 h. TRPV4-GFP protein, affinity-purified from total cell lysates, was separated by SDS-PAGE and stained with Coomassie blue dye, followed by gel band excision and digestion with trypsin. The resulting peptide mixture was separated by liquid chromatography on a C18 reverse-phase column over an acetonitrile gradient and then analyzed by tandem mass spectrometry. *A* and *B*, MS/MS spectra of HCAEC pS823 and pS824, in which one of the double-charged (2+) precursor peptides selected by MS1, with m/z of 591.2 for pS823 and 455.7 for pS824, respectively, was fragmented by MS2. The resulting peaks in the MS/MS spectrum correspond to a series of b and y ions resulting from the fragmentation of a peptide containing phosphorylated TRPV4 S823 or S824, respectively. *C* and *D*, similar MS/MS spectra of pS823 and pS824 peptides were obtained in TRPV4-GFP purified from HEK 293 cells.

HCAECs were transduced with plasmids encoding human TRPV4-GFP in a lentiviral vector and HEK 293 cells transfected with TRPV4-GFP plasmids by lipofectamine. TRPV4-GFP proteins were affinity-purified by anti-GFP magnetic beads and then used for the identification of phosphorylation sites by mass spectroscopy. We detected phosphorylation at either the first or second serine of the trypsin-digested peptide WSSVVPR in both cell lines, which corresponds to Ser-823 and Ser-824 in hTRPV4, respectively (Fig. 1, *A* and *B*). Double phosphorylation at Ser-823 and Ser-824 were not detected. Since the cells were not pre-stimulated with protein kinase activators, the phosphorylation detected at these two sites likely represents basal levels of phosphorylation in HCAECs and HEK 293 cells. Protein alignment of TRPV4 homologs indicates that the corresponding Ser-823 residue is highly conserved and can be seen in most of the species other than *Caenorhabditis elegans*, as compared to Ser-824 which differs with selected organisms (54). Thus, Ser-823 is evolutionarily conserved in nature between TRPV4 channels of different species.

**Figure 2:**
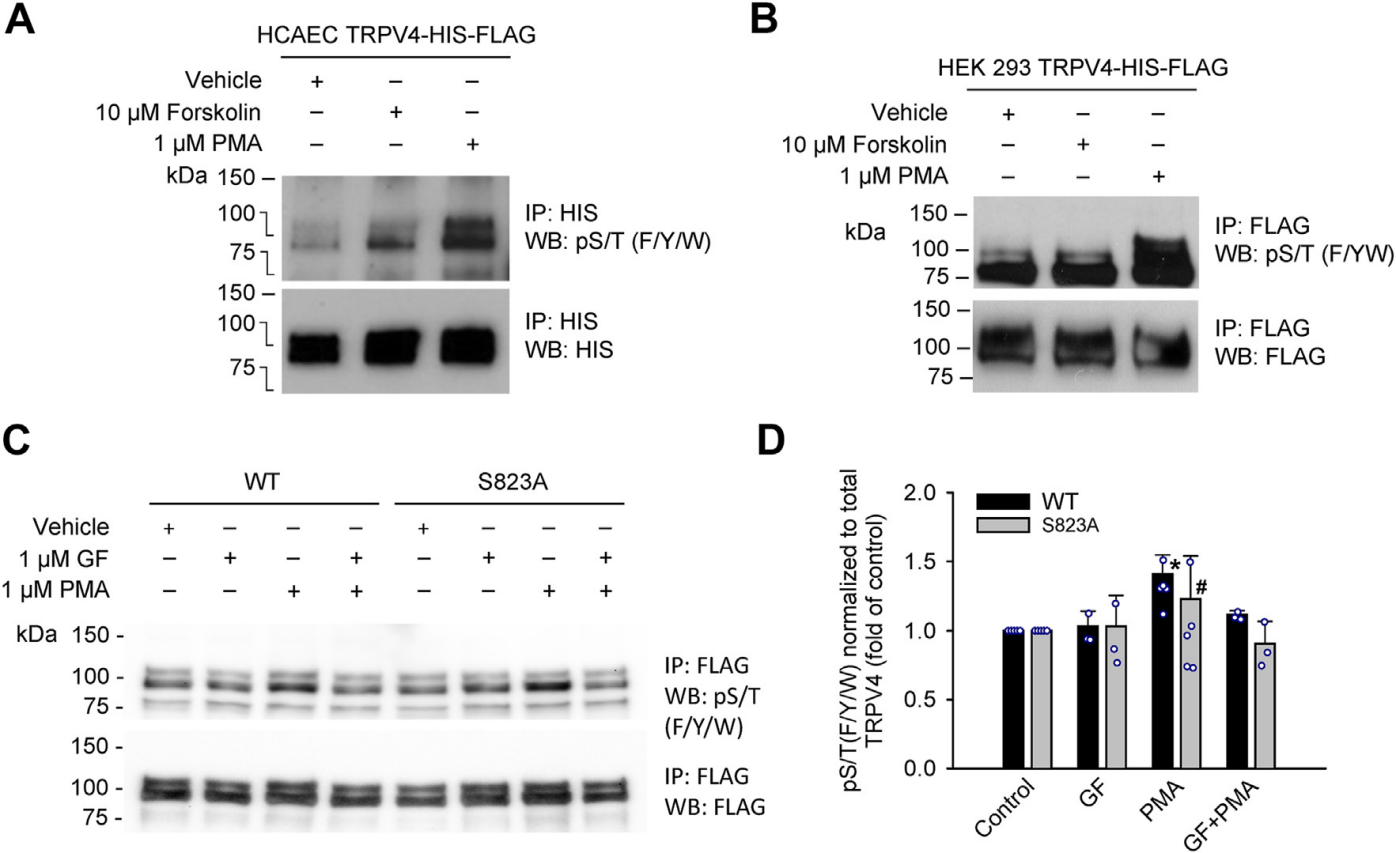
**TRPV4 phosphorylation in response to PKA and PKC activation.***A* and *B,* HCAECs were transduced with plasmids encoding TRPV4-HIS-FLAG in a lentiviral vector, and HEK 293 cells transfected with TRPV4-HIS-FLAG plasmids by lipofectamine. Cells were treated with PKA activator forskolin (10 μM) or PKC activator PMA (1 μM) for 30 min. After immunoprecipitation with anti-HIS or anti-FLAG antibodies, TRPV4 phosphorylation at Ser-823 or other unknown sites was analyzed by Western blotting using a phospho-(Ser/Thr) motif antibody that detects phospho-serine or threonine in the context of phenylalanine, tyrosine, or tryptophan at the −1 position [(F/Y/W) (S∗T∗)] or phenylalanine at the +1 position [(S∗T∗)F]. For total TRPV4 detection, the same membrane was reprobed with anti-HIS antibodies for HCAEC or anti-FLAG antibodies for HEK 293 cells. *C*, HEK 293 cells transfected with TRPV4-HIS-FLAG WT or S823A plasmids were treated with PKC inhibitor GF 109203X (1 μM) for 30 min, PKC agonist PMA (1 μM) for 30 min, or a combination of GF 109203X and PMA for 30 min each. TRPV4 phosphorylation was analyzed with a similar method as in *A* and *B*. Immunoblots are representative of (*A*) two (n = 2), (*B*) two (n = 2), and (*C*) five independent experiments (n = 5). *D*, quantification of TRPV4 phosphorylation. Data represent mean ± S.D. of pS/T(F/Y/W) band densities normalized to TRPV4 expression levels and then converted to fold changes as compared with control. ^∗^*p* < 0.05 vs control; ^#^*p* < 0.05 vs WT PMA (two-way ANOVA).

Next, we performed Western blotting analysis to further determine the phosphorylation of serine and threonine residues in TRPV4. For this analysis, HCAECs were transduced with plasmids encoding TRPV4-HIS-FLAG in a lentiviral vector, and HEK 293 cells were transfected with TRPV4-HIS-FLAG plasmids by lipofectamine. Cells were treated with vehicle, PKA activator forskolin (10 μM), or PKC activator phorbol 12-myristate 13-acetate (PMA; 1 μM) for 30 min. After immunoprecipitation with anti-HIS or anti-FLAG antibodies, TRPV4 phosphorylation at Ser-823 or other unidentified S/T residues was examined with a phospho-(Ser/Thr) motif antibody that detects phospho-serine or threonine in the context of phenylalanine, tyrosine, or tryptophan at the −1 position [(F/Y/W) (S∗/T∗)] or phenylalanine at the +1 position [(S∗/T∗)F]. As shown in Figure 2, *A* and *B*, we found that treatment of HCAECs and HEK 293 cells with 1 μM PMA resulted in a marked increase of phosphorylation in TRPV4 as compared to vehicle-treated control. In contrast, forskolin only slightly increased TRPV4 phosphorylation in HCAECs but did not significantly affect TRPV4 phosphorylation in HEK 293 cells. These results suggest that the corresponding pS/T(F/Y/W) residue(s) in TRPV4 is mainly phosphorylated by PKC, although it can also be phosphorylated to a smaller extent by PKA in HCAECs. The S823 resides in one of the canonical motifs (RXS∗/T∗) recognized by the basophilic AGC kinases such as PKA and PKC. Further, an unknown phosphorylated protein at 75 kDa was observed to co-immunoprecipate with TRPV4, the identity of which is to be explored in future studies.

To confirm the specificity of PKC to Ser-823 residue, HEK 293 cells were transfected with TRPV4-HIS-FLAG WT or S823A plasmids and then treated with PKC agonist PMA (1 μM) for 30 min in the absence or presence of PKC inhibitor GF 109203X (1 μM) for 30 min. TRPV4 phosphorylation at Ser-823 or other unidentified S/T residues was analyzed with a similar method as described above. As shown in Figure 2, *C* and *D*, the PMA-induced increase in S/T phosphorylation of WT TRPV4 was significantly inhibited by GF 109203X. In the non-phosphorylatable mutant (S823A), PMA induced less increase in the S/T phosphorylation of TRPV4 after normalization to total TRPV4, indicating that Ser-823 is at least partially involved in TRPV4 S/T phosphorylation by PKC pathway.

The phosphorylation of TRPV4 has been examined by previous studies using a similar protein overexpression approach in HEK293 or other cell lines, as well as in primary cells with endogenous TRPV4 such as Madlin-Darby canine kidney (MDCK) cells and HCAECs (54, 55). Consistent with the reported lack of endogenous expression of TRPV4 in HEK293 cells (56, 57) or a very low level of TRPV4 in some batches of HEK293 cells (58), we did not detect a significant amount of endogenous TRPV4 protein in HEK293 cells (Fig. S1*A*). HCAECs do express endogenous TRPV4 at lower levels, whereas the expression of TRPV4 in transfected HCAECs is ∼15-fold higher compared to non-transfected HCAECs (Fig. S1*B*, lower blot). However, the phosphorylation of endogenous TRPV4 (*e.g.*, pS824) was not readily detectable in non-transfected HCAECs, at least under the current experimental conditions (Fig. S1*B*, upper blot).

### Ser-823 and Ser-824 phosphorylation contribute to the regulation of TRPV4 activation by AA in HCAECs

**Figure 3:**
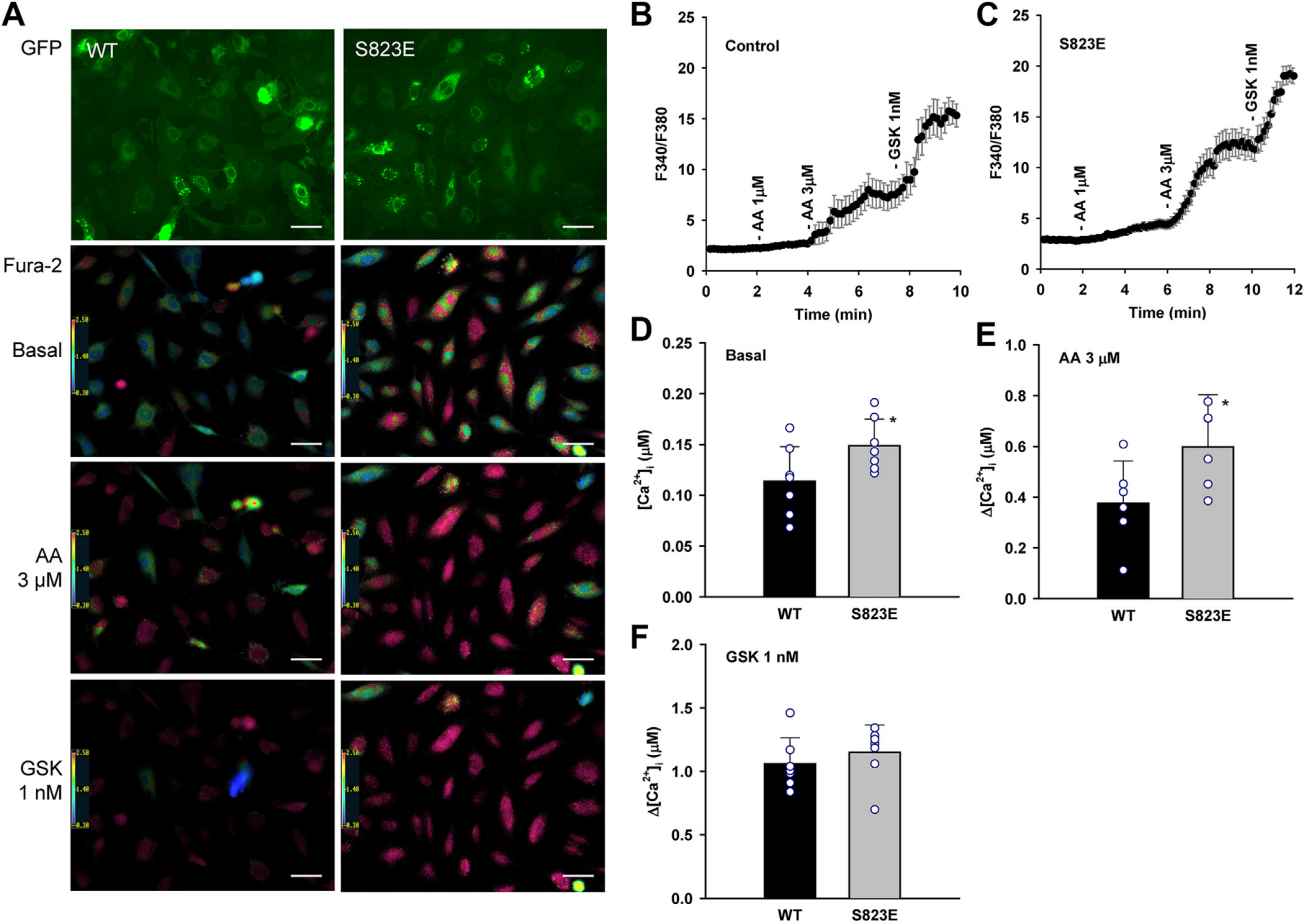
**Effect of S823E substitution on arachidonic acid- and GSK1016790A-induced TRPV4 activation in HCAECs.***A*, representative images of fura-2 calcium assay of HCAECs transduced with wildtype (WT) or S823E mutant TRPV4-GFP in a lentiviral vector. Cells were treated with endogenous TRPV4 activator arachidonic acid (AA; 3 μM), followed by synthetic TRPV4 agonist GSK1016790A (1 nM). The F340/F380 ratio is set on a scale of 0.3 to 2.5, with the corresponding color changes from *blue* to *red*. Scale bar, 50 μm. *B* and *C*, representative traces of fura-2 calcium assay where the *black* line denotes mean F340/380 ratios and error bars 1 × S.E. *D–F*, summarized data for basal intracellular calcium concentrations ([Ca^2+^]_i_) in WT and S823E mutants, AA- and GSK1016790A-induced Ca^2+^ increase (Δ[Ca^2+^]_i_ from baseline), respectively. All data represent mean ± S.D. with the data points from each independent experiment super-imposed on the bar graph; n = 7 (*D*), 6 (*E*), and 7 (*F*) independent experiments. ∗*p* < 0.05 compared with WT.

To determine the role of S823 phosphorylation in TRPV4 activation by AA, an endogenous lipid activator, we analyzed TRPV4-dependent Ca^2+^ influx using ratiometric fura-2 calcium imaging in HCAECs transduced with the following TRPV4 constructs: wildtype (WT), phosphomimic mutant (S823E), and non-phosphorylatable mutants (S823A, S824A and S823A-S824A). As shown in Figure 3, the basal intracellular calcium concentration ([Ca^2+^]_i_) of the phosphomimic S823E mutant was increased compared with the WT. Furthermore, a significantly enhanced response to AA (3 μM) was observed in the S823E mutant (Δ[Ca^2+^]_i_) compared with the WT TRPV4. In contrast, the response to the synthetic chemical agonist GSK1016790A (1 nM) was similar in the S823E mutant compared with the WT TRPV4. These results suggest that Ser-823 phosphorylation increases basal channel activity and enhances TRPV4 activation by AA, similar to Ser-824 phosphorylation reported previously (54).

**Figure 4:**
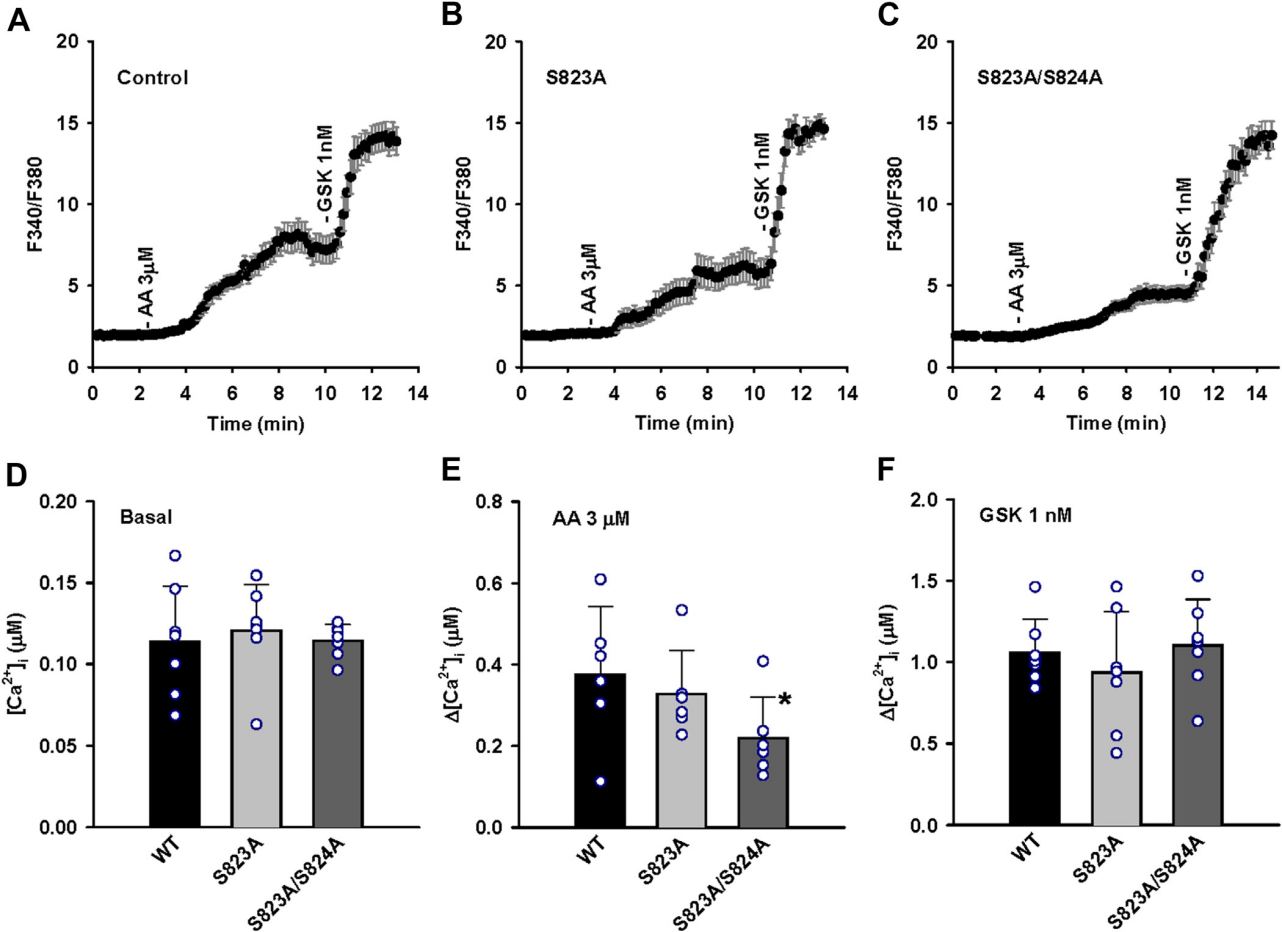
**Effect of S823A and S823A/S824A substitutions on arachidonic acid- and GSK1016790A-induced TRPV4 activation in HCAECs.** Fura-2 calcium assay of HCAECs transduced with wildtype (WT), S823A, or S823A/824A mutant TRPV4-GFP in a lentiviral vector. Cells were treated with endogenous TRPV4 activator arachidonic acid (AA; 3 μM), followed by synthetic TRPV4 agonist GSK1016790A (1 nM). *A–C*, representative traces of fura-2 calcium assay where the *black* line denotes mean F340/380 ratios and error bars 1 × S.E. *D–F*, summarized data for basal intracellular calcium concentrations ([Ca^2+^]_i_) in WT, S823A, and 823A/824A mutants, and AA- and GSK1016790A-induced Ca^2+^ increase (Δ[Ca^2+^]_i_ from baseline), respectively. All data represent mean ± S.D. with the data points from each independent experiment super-imposed on the bar graph; n = 7 (*D*), 6 (*E*), and 7 (*F*) independent experiments. ∗*p* < 0.05 compared with WT.

Compared with the WT TRPV4, AA-induced Ca^2+^ response was slightly lower in HCAECs expressing the S823A mutant compared to WT, however, the response was significantly blunted in HCAECs expressing the S823A/S824A mutant (Fig. 4). Subsequent addition of GSK1016790A (1 nM) increased [Ca^2+^]_i_ to similar levels in S823A-S824A mutant and to slightly lower levels in the S823A mutant. The basal [Ca^2+^]_i_ was comparable in the HCAECs expressing WT, S823A, and S823A-S824A mutants.

### TRPV4 S823A and S823E mutants localize on the cell surface of HCAECs

**Figure 5:**
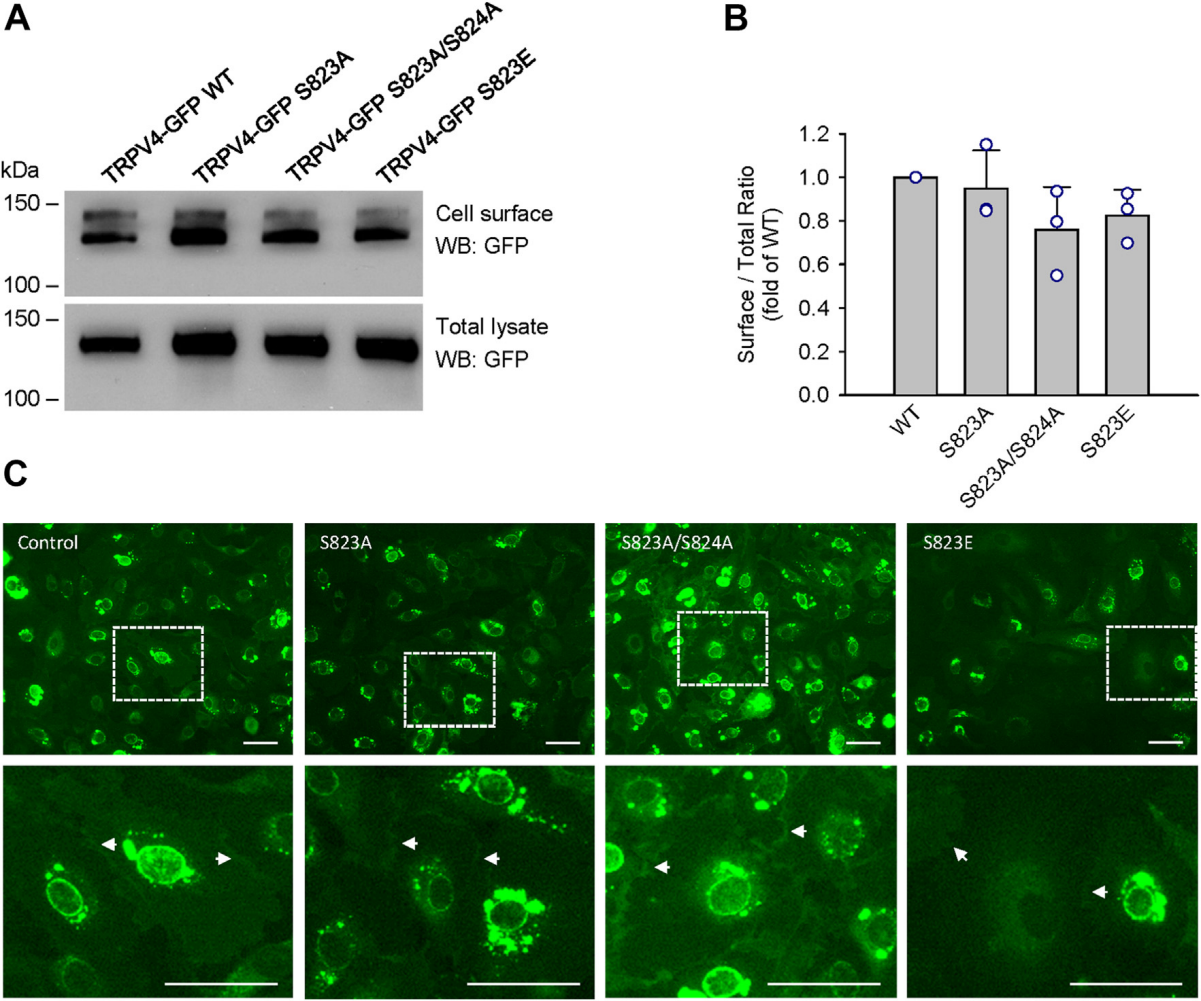
**TRPV4 S823A, S823A/S824A, and S823E mutants are localized to the plasma membrane of HCAEC cells.***A*, Western blots with GFP antibodies of cell surface and total TRPV4-GFP WT, S823A, S823A/S824A, or S823E mutant proteins expressed in HCAECs. Cell surface proteins were labeled using a cell surface biotinylation method and captured with NeutrAvidin agarose beads. Total cellular lysates were analyzed in parallel. *B*, ratios of the cell surface to total levels of TRPV4 protein for S823A, S823A/S824A, and S823E mutants as compared with WT TRPV4 (mean ± S.D., *p* > 0.05, n = 3). *C*, representative fluorescence images of HCAECs expressing TRPV4-GFP WT, S823A, S823A/S824A, or S823E mutants. White arrows indicate plasma membrane localization of TRPV4 proteins. Scale bar, 50 μm. Data in *C* are representative of at least five independent experiments.

Expression of TRPV4 channels on the cell membrane of HCAECs is necessary to determine their functionality in these cells. To determine whether S823A, S823A/S824A, and S823E mutations affect the trafficking to the cell surface, quantitative analysis of cell surface expression was performed by biotinylation labeling followed by Western blot. Results obtained confirmed the expression of WT and mutant TRPV4 GFP fusion proteins on the plasma membrane (Fig. 5, *A* and *B*). Additionally, fluorescence imaging of HCAECs expressing TRPV4-GFP fusion proteins (WT, S823A, S823A/S824A, and S823E) was performed during the fura-2 calcium assay. The fluorescence images revealed the protein expressions of fusion proteins of both WT and mutants on the cell surface (Fig. 5*C*).

### PKA and PKC phosphorylation contribute to the regulation of TRPV4 activation by AA

**Figure 6:**
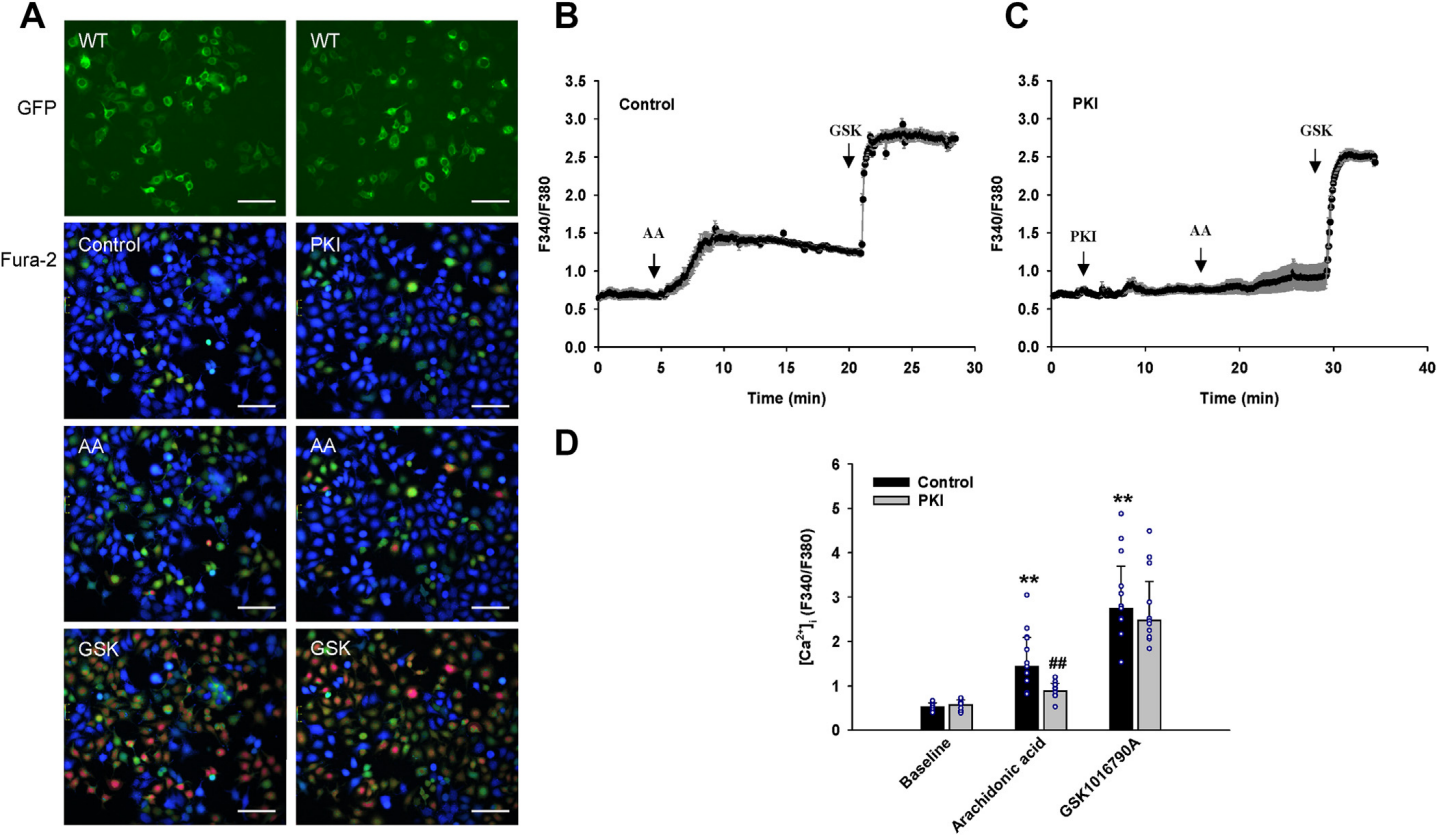
**Inhibitory effect of PKI 14–22, a protein kinase A inhibitor, on arachidonic acid-induced TRPV4 activation in HEK 293 cells transiently expressing TRPV4-GFP.***A*, representative images of fura-2 calcium assay of HEK 293 cells transfected with wildtype (WT) TRPV4-GFP plasmids. Cells were stimulated with arachidonic acid (AA; 6 μM), followed by synthetic agonist GSK1016790A (GSK; 10 nM) without or with pretreatment with PKI 14-22 (PKI, 1 μM). Scale bar, 100 μm. *B* and *C*, representative traces of fura-2 calcium assay where the *black* line denotes mean F340/380 ratios and the error bars × S.E. *D*, summarized data for intracellular calcium concentrations ([Ca^2+^]_i_) in control (n = 10) and PKI-treated (n = 9) HEK 293 cells. All data represent mean ± S.D. ^∗∗^*p* < 0.01 compared with baseline, ^##^*p* < 0.01 vs compared with control (two-way ANOVA).

**Figure 7:**
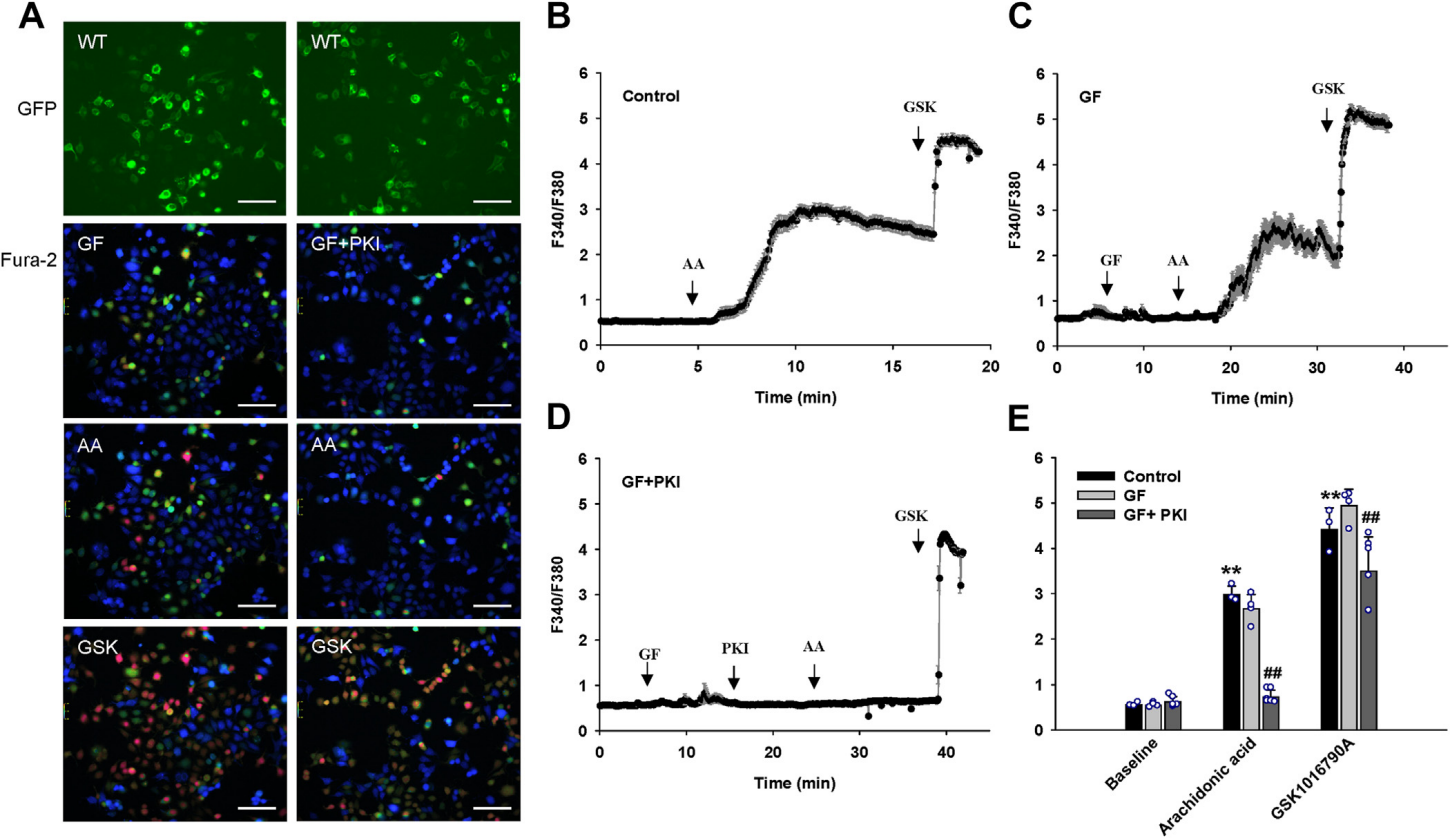
**Effect of GF 109203X, a protein kinase C inhibitor, alone and in combination with PKI 14–22 on arachidonic acid-induced TRPV4 activation in HEK 293 cells transiently expressing TRPV4-GFP.***A*, representative images of fura-2 calcium assay of HEK 293 cells transfected with wildtype (WT) TRPV4-GFP plasmids. Cells were stimulated with arachidonic acid (AA; 6 μM), followed by synthetic agonist GSK1016790A (GSK; 10 nM) after pretreatment with GF 109203X (GF, 1 μM) alone or in combination with PKI 14-22 (PKI; 1 μM). Scale bar, 50 μm. *B*–*D*, representative traces of fura-2 calcium assay where the *black* line denotes mean F340/380 ratios and the error bars 1 × S.E. *E*, summarized data for intracellular calcium concentrations ([Ca^2+^]_i_) in control (n = 3), GF-treated (n = 4), and GF+PKI-treated (n = 6) HEK 293 cells. All data represent mean ± S.D. ^∗∗^*p* < 0.01 compared with baseline, ^##^*p* < 0.01 vs compared with control (two-way ANOVA).

To confirm the role of PKA and PKC in the activation of TRPV4 by AA, we performed calcium imaging studies after inhibiting the phosphorylation of TRPV4 by PKI 14-22 (PKA inhibitor, myristoylated) and GF 109203X (PKC inhibitor). As shown in Figure 6, AA- but not GSK1016790A-induced Ca^2+^ response of HEK 293 cells expressing WT TRPV4 were markedly reduced by PKI 14-22. However, AA-induced Ca^2+^ response was only slightly reduced by GF 109203X while the response was eliminated by a combination of both GF 109203X and PKI 14-22 (Fig. 7). Subsequent addition of GSK1016790A (10 nM) increased the [Ca^2+^]_i_ to similar levels in control and GF 109203X-treated cells but to a slightly lower level in cells treated with both GF 109203X and PKI 14-22 (Fig. 7).

To further characterize TRPV4 regulation by PKA and PKC, we analyzed the TRPV4-expressing HEK 293 cells with high basal [Ca^2+^]_i_ (F340/F380 > 1.0) since the effects of PKA and PKC inhibitors could be readily seen in those cells (Figs. S2 and S3). When applied individually, PKI 14-22 and GF 109203X rapidly reduced the basal [Ca^2+^]_i_ but had much less effect on the subsequent response to AA. However, PKI in combination with GF 109203X eliminated the response to AA as seen in cells with low basal [Ca^2+^]_i_ (F340/F380 < 1.0). Subsequent addition of GSK1016790A (10 nM) increased the [Ca^2+^]_i_ to similar levels in control, PKI 14-22-, and GF 109203X-treated cells; however, a slight reduction in the response to GSK1016790A was seen in cells when PKI 14-22 and GF 109203X were used in combination.

### A refined homology model at the C-terminal helix of the TRPV4 channel

**Figure 8:**
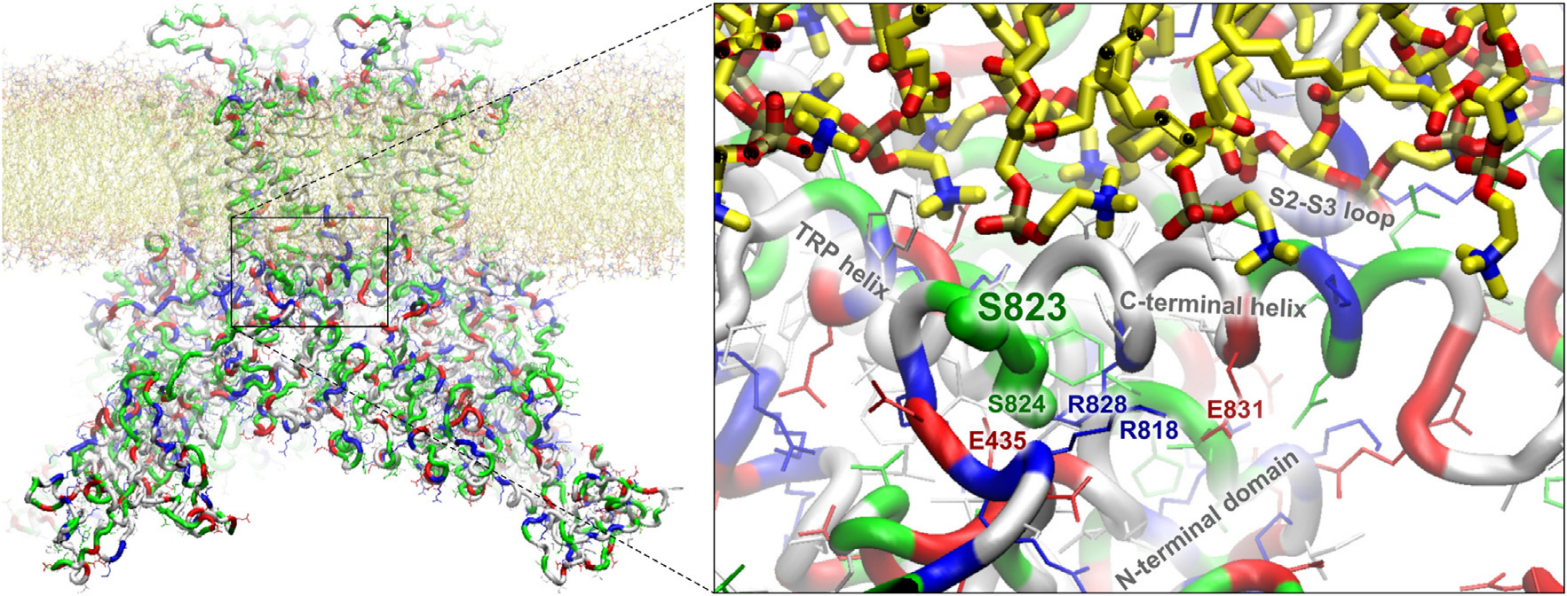
**A refined homology model of TRPV4 illustrating the putative docked position of the C-terminal helix and possible ways for S823 and S824 phosphorylation to dislodge it.** The *left panel* shows a whole-channel view of the simulated refined model, while the *right panel* zooms in on the proposed docked location of the C-terminal helix. Membrane lipids have carbons colored *yellow*, oxygens *red*, phosphorus tan, and *nitrogen blue*. The protein backbone is presented as a “tube”, colored by residue type (aliphatic residues *white*, *polar green*, basic *blue*, and acidic *red*). The side chains, visible on the zoom-in, are shown as thin bonds, colored the same way. The side chains for S823 and S824 are shown as a thick “licorice”. The docked helix is stabilized by a combination of hydrophobic interactions and several salt bridges between the charged residues of the domain and the channel. S823 can be accessed from the outside with no major salt bridges around except a few phosphate groups seen near the hydroxyl group of S823. Ser-823 phosphorylation could prevent the docking and sensitize the TRPV4 activation by AA by pushing the helix away from the bilayer when there is an increase in negatively charged lipids.

To gain insights into the mechanism of C-terminal regulation, we performed homology modeling of human TRPV4 at the C-terminal helix and discussed the possible ways to dislodge the helix during the phosphorylation of S823 and S824 for channel activation in response to AA. Our previously published full-length homology model of TRPV4 (54) has suggested a putative structure and docking location for the C-terminal alpha-helix and provided a consistent hypothesis explaining the effect of S824 phosphorylation on channel function. However, that model was based on the homology to the TRPV2 structure as no TRPV4 channels were resolved by that time. In this study, we refined our original model by using the TRPV4 structure (PDB ID 6BBJ) as a template with an older model docked C-terminal helix (still unresolved experimentally today) as the starting conformation (Fig. 8). After the steered transformation, of the backbone of the old model to the resolved position in the 6BBJ structure, the new model was refined through repeated cycles of unrestrained simulations and symmetry-driven simulated annealings (see Experimental procedures for details). The overall topology of the channel and the docking position of the C-terminal helix remained very similar, with minor adjustments, suggesting the stability of the arrangement.

## Discussion

The cytosolic C-terminal domain of TRPV4 is considered to be important for channel protein assembly, trafficking, and other regulatory functions (40, 41). Several amino acid residues of the C- terminal tail have been proposed as candidate substrates for serine/threonine and tyrosine kinase-dependent phosphorylation but only two phosphorylation sites (Ser-824 and Tyr-805) have been unambiguously identified till now by mass spectrometry or motif-specific antibodies (50, 51, 52, 53, 54). Among those two identified residues, phosphorylation at Ser-824 sensitizes TRPV4 to selected stimuli such as hypotonic cell swelling and AA while the functional significance of Tyr-805 phosphorylation remains unclear (52, 55). In the present study, we identified the neighboring residue of Ser-824, *i.e.*, Ser-823, as an alternative phosphorylation site involved in the regulation of TRPV4 activation by the lipid mediator AA.

### Regulation of AA-induced TRPV4 activation by PKA and PKC through Ser-823/Ser-824 phosphorylation

Protein post-translational modifications of the TRPV4 channel such as ubiquitination, glycosylation, and phosphorylation have been extensively studied for their pivotal role in regulating the channel activity (59, 60, 61), including the phosphorylation of specific residues in the N and C-termini (52, 53, 54, 55) Identification of the TRPV4 phosphorylation using an unbiased mass spectrometry-based proteomic approach is often performed by stimulating cells with protein kinase activators, *e.g.*, Ser-824 by PMA (51); however, only a few residues such as Tyr-110 and Tyr-805 (55) were detected at basal levels. We found that Ser-824 and Ser-823 basal phosphorylation could be detected by mass spectroscopy in both HCAECs and HEK 293 cells, suggesting that S823, a new phosphorylation site in the cytosolic C-terminal tail of TRPV4, is important for channel physiology besides the well-known Ser-824 residue. However, the detection of endogenous TRPV4 phosphorylation by mass spectrometry or Western Blot was not possible due to the minimal expression of TRPV4 channels in these cells (Fig. S1).

Protein phosphorylation by different kinases has been observed in TRPV4, for example, Ser-162/Thr-175/Ser-189 phosphorylation by PKC, and Ser-824 phosphorylation by PKA, PKC, and SGK kinases (50, 54, 62). Mutations of these phosphorylation sites to alanines reduced phosphorylation levels and consequently diminished the TRPV4 responses to TRPV4 stimuli such as hypotonic cell swelling and the lipid mediator AA (50, 51, 54). Our study is consistent with these previous observations in that protein phosphorylation of N- and C-terminal residues sensitizes or is required for TRPV4 activation by selected channel stimuli. Interestingly, we also found that Ser-823 phosphorylation was preferentially stimulated by PMA (PKC activator) than forskolin (PKA pathway activator). However, it remains likely that other unidentified residue(s) also contributes to the phosphorylation of TRPV4 channels under the current experimental conditions. In addition, the variability introduced by the mutant should be considered because of the increase in total TRPV4 expression in PMA-stimulated S823A mutants. Previously, we found that forskolin-induced Ser-824 phosphorylation was enhanced in the presence of cantharidin (protein phosphatase 1/2A inhibitor) (54); however, Ser-823 phosphorylation did not change significantly in the presence of cantharidin, indicating that PP1/2A does not regulate Ser-823 phosphorylation catalyzed by the PKA.

The intracellular localization of WT and S823A/E mutant TRPV4 channels on the HCAECs were comparable in terms of the overall cell surface expression of TRPV4-GFP and additional retention in the endoplasmic reticulum (ER), as seen previously in TRPV4-transfected HEK293 cells (63). It is of note that specific C-terminal domains of TRPV4 such as the 828 to 844 region are required for plasma membrane localization and mutation-induced retention of TRPV4 in the ER can be partially rescued by endogenous TRPV4 (41). Therefore, it is possible that the observed channel activities of TRPV4 WT or mutants, a tetrameric protein, may derive from endogenous TRPV4 protein expressed in a cell type studied. In the present study, we found that HCAECs, but not HEK 293 cells, do express endogenous TRPV4 proteins but only at minimal levels as compared with TRPV4-overexpression cells, which makes it less likely to form significant levels of mixed tetramers with exogenous TRPV4. Furthermore, in non-transfected HCAECs, calcium responses to AA and GSK10167890A is negligible according to our previous studies (23).

The specific roles of various protein kinases and phosphorylation sites in the regulation of TRPV4 channels in response to defined endogenous channel activators such as the lipid mediator AA are not well understood. Our findings of only slightly reduced responses to AA in S823A mutant compared to WT but much greater inhibition of AA-induced responses in S823A-S824A double mutant suggests that the effect of Ser-824 phosphorylation likely predominates under the basal conditions. Studies with PKA and PKC inhibitors also support the hypothesis of the minor role of S823 phosphorylation by PKC, where AA-induced TRPV4 activation was significantly reduced by the PKA pathway inhibitor and slightly attenuated by the PKC pathway inhibitor, while the response was eliminated after inhibiting both pathways. Collectively, these results suggest that PKA plays a major role in the regulation of TRPV4 activation in response to AA while PKC may play a secondary role in AA-induced TRPV4 activation but can become an important regulator of TRPV4 function under different conditions such as elevated intracellular [Ca^2+^]_i_ in cells. Interestingly, a slight increase in basal Ca^2+^ levels was observed in the S823E mutant without apparent cytotoxicity, which is in contrast to some pathological mutations involved in neurological and skeletal muscle channelopathies (37, 64, 65).

### Regulation of TRPV4 activation by N- and C-terminal domains

The mechanisms by which TRPV4 is activated by diverse physical and chemical stimuli remain poorly understood but the current literature indicates at least two independent pathways of activation. First, synthetic agonists such as phorbol ester 4α-PDD has been proposed to activate TRPV4 by directly binding to its TM3 and TM4 segments of the channel (18), which is largely supported by recent cryo-EM structures of TRPV4 in complex with this compound (37, 38). Second, TRPV4 activation in response to hypotonic cell swelling, high viscous load, and shear stress appears to depend on phospholipase A_2_ (PLA_2_)-catalyzed release of AA and its metabolites (66, 67). Consistent with this proposal on two independent pathways of activation, we also found that Ser-823/Ser-824 phosphorylation preferentially affected TRPV4 activation by AA but not by the synthetic agonist GSK1016790A.

Previous studies have shown that the cytosolic N-terminal domain also regulates various functional aspects of TRPV4, especially responses to lipid molecules such as PIP_2_ (20, 68, 69). The N-terminal linker region also harbors the previously postulated arachidonate recognition sequence (ARS) (21) as illustrated in our hypothetical model of AA-induced TRPV4 activation and its modulation by Ser-824 phosphorylation (54). This putative role of ARS in the ligand-receptor-like interaction between AA and TRPV4 is supported by the findings that several fatty acids, saturated and poly-unsaturated fatty acids (PUFAs) bind to these channels, but do not activate/regulate as well as AA (unpublished observations), likely due to the difference in their specificities for binding and activation at molecular levels as seen in fatty acid binding GPCRs and AA metabolizing enzymes such as cyclooxygenase (70).

Similar to N-terminal regulation, CaM binding domain (CBD) situated in the C-termini of the channel (where Ser-823 and Ser-824 reside) has been implicated in the Ca^2+^/CaM regulation of the channel and, mutants lacking CBD have shown increased channel activity (40, 42, 71, 72), whereas deletion of other residues in the C-terminus (*e.g.*, 838–857) inhibited the formation of functional channels. Our previous studies on Ser-824 phosphorylation have provided insight into the possible mechanisms of TRPV4 channel opening and regulation by phosphorylation and these results further allowed us to decipher other possible phosphorylation sites (such as Ser-823) involved in the regulation of channel opening. Given its complex polymodal gating behavior, identifying these different modalities and the residues involved in the activation/regulation of TRPV4 will be necessary to define the underlying mechanisms of this channel.

### Structural interpretation of the experimental findings using a refined homology model at the C-terminal helix of the TRPV4 channel

As extensively discussed in our previous homology model of TRPV4 (54), the hypothetical C-terminal helix is docked in a crevice between the cytoplasmic N-terminal domain and S2-S3 loop of the transmembrane domain, in the vicinity of the crucial TRP helix (Fig. 8). Dislodging the C-terminal helix would facilitate the outward movement of the TRP helix in response to certain stimuli, including AA and tension (54, 73, 74). The position of the docked helix is stabilized by a combination of hydrophobic interactions and several (up to 6) salt bridges between the charged residues of the domain and the rest of the channel. Specifically, phosphorylation of the partially buried S824 residue (54) would likely disrupt (by intercepting the basic residues) the bridges between R828 and E435, as well as R818 to E831 thus destabilizing the docked position. In contrast, the position of S823 is more accessible from the outside and not in the vicinity of the major salt bridges, however, the hydroxyl of S823 is frequently seen close to the phosphate groups of the lipid bilayer. Phosphorylation of S823 (*i.e.* introduction of a bulky negatively charged group) would likely push the helix away from the bilayer and dislodge it from the docked location. A testable prediction would be that with the increase of the negatively charged lipids in the membrane, phosphorylation of S823 would be more effective in the activation of the channel. We recognize that several Cryo-EM structures of human TRPV4 have been recently published by our collaborator and others (37, 38) shortly after the development of our refined model of TRPV4 regulation by C-terminal phosphorylation. Although an updated model based on the new structure of human TRPV4 would be warranted for future studies, our current model and the new structure are quite similar in the proposed docking region of the C-terminal helix and, thus an updated model does not change the structural interpretation of the experimental findings in the current study.

Given that the locations of S823 and especially S824 are not very easily accessible for relatively bulky kinases, one can assume that for a WT TRPV4, there is some sort of quick dynamic equilibrium with partial undocking of the C-terminal helix, that is long enough for engaging into interactions with kinases to get phosphorylated, but not long enough for the whole process of binding AA by TRPV4 and opening of the channel. However, this hypothesis requires further testing in future studies considering that our refined model of TRPV4 regulation by C-terminal phosphorylation (Fig. 8) remains hypothetical and the structure and the position of the C-terminal helix has to be modeled until it is eventually resolved in a structure experimentally.

In summary, we present evidence in support of TRPV4 phosphorylation at Ser-823 by the 10.13039/100012258PKC pathway and its involvement in the channel activation by the endogenous lipid mediator AA. Through the phosphorylation of discrete residues such as Ser-823, Ser-824, or others, both PKA and PKC thus contribute to the regulation of TRPV4 activation in a stimulus-specific manner. Our study also provides insight into the potential structural mechanisms by which the phosphorylation of the cytosolic C-terminal domain regulates the activation of TRPV4 channels.

## Experimental procedures

### HEK 293 and HCAEC culture

HEK 293 cells provided by Dr David Wilcox (Medical College of Wisconsin) were maintained in DMEM Medium (Gibco) supplemented with 10% FBS and 1% PSG at 37 °C and 7.5% CO_2_. Cells between passages 11 to 15 were used for experiments. HCAECs obtained from Lonza (Walkersville, MD) were maintained in a complete EGM-2MV growth medium at 37 °C and 5% CO_2_ according to the manufacturer’s protocol. HCAECs were split at a 1:3 ratio once the cells reached 90 to 95% confluency.

### Plasmids, mutagenesis, and lentiviral production

The full-length human TRPV4 (NM_021625) with its C-terminus tagged with turbo GFP, or N-terminus tagged with HIS-DDK tag was constructed in a lentiviral plasmid vector as reported previously (23, 54). Point mutations S823A, S823E, and S823A/S824A were introduced into wildtype TRPV4 by using site-directed mutagenesis. All constructs were verified by DNA sequencing and lentiviruses were produced from HEK 293 cells as described previously (54, 75).

### Transient and stable TRPV4 transgene expression

For stable TRPV4 overexpression experiments, HCAECs at passage six were grown to 50 to 60% confluence before being transduced with recombinant lentiviruses at an m.o.i. (multiplicity of infection) of 5 to 10. 16 hours after transduction, the concentration of free calcium in the culture medium was reduced to ∼0.6 mM by the addition of 1.2 mM EDTA, and the medium pH was readjusted. to lower the toxicity and calcium overload due to the TRPV4 overexpression (23). Cells were used for calcium imaging 3 to 4 days after transduction (without further cell splitting).

HEK 293 cells were transfected with TRPV4 plasmids using Lipofectamine 2000 reagent (Invitrogen) in Opti-MEM for 3 h (calcium imaging) or 6 h (immunoblotting experiments). Cells were then incubated in a complete DMEM medium with reduced Ca^2+^ (0.6 mM) for an additional 16 to 20 h before calcium imaging, or 48 h before immunoblotting experiments. In some experiments that require more TRPV4 proteins (Fig. 2, *B* and *C*), HC067047 (1–2 μM) was added in the low-calcium medium after 6 h of transfection to further reduce the potential toxicity associated with high-level expression of TRPV4 in HEK293 cells.

### Calcium imaging

HCAECs or HEK 293 cells were plated onto 35-mm glass-bottom Petri dishes and grown to 70 to 80% confluence. The cells were incubated with fura-2 AM (5 μM) in the presence of Pluronic F-127 (0.01%) at 37^o^C for 45 min in the respective medium. After the incubation, cells were washed with a modified Hanks’ balanced salt solution (HBSS) containing (in mM): 123 NaCl, 5.4 KCl, 1.6 CaCl_2_, 0.5 MgCl_2_, 0.4 MgSO_4_, 4.2 NaHCO_3_, 0.3 NaH_2_PO_4_, 0.4 KH_2_PO_4_, 5.5 glucose and 20 HEPES (pH 7.4 with NaOH). Meta Fluor software was used for recording and analyzing the cytosolic Ca^2+^ signals emitted as fura-2 fluorescence at 510 nm in cells exposed to alternate 340 and 380 nm excitation wavelengths (54). Unless otherwise stated, we excluded the cells with very high basal [Ca^2+^]_i_ (F340/F380 > 3.0 and >1.0 for TRPV4-expressing HCAECs and HEK 293, respectively) during the data analysis. This approach has been used previously to minimize potential variations in response to TRPV4 agonists (51, 54).

### Immunoprecipitation and Western blotting

HCAECs and HEK 293 cells expressing the TRPV4 transgene (wild-type and mutant 823A) were rinsed with ice-cold phosphate-buffered saline (PBS, pH 7.4), and lysed with buffer containing 1× Modified Dulbecco’s PBS Buffer as a base buffer (D-PBS; Thermo Scientific, Cat No: 28344) containing 8 mM sodium phosphate, 2 mM potassium phosphate, 0.14 M NaCl, 10 mM KCl, pH 7.4, supplemented with 1% Triton X-100, 10 μM cantharidin, 20 mM N-ethylmaleimide (NEM), and a mixture of 1× protease (Roche) and 1× phosphatase inhibitors (Pierce). Cell lysates were centrifuged at 12,000*g* for 10 min, and protein samples (100–200 μg) were mixed with 0.5 μg of anti-FLAG M2 antibody (F1804-200 UG; Sigma Aldrich) for 3 h and then with 5 μl of protein A/G magnetic beads (Pierce, cat. no. 88802) overnight at 4 °C to precipitate protein immunocomplexes. The beads were then washed 3 times with the IP buffer (D-PBS-1% Triton X-100, a mixture of protease and phosphatase inhibitors) and once with D-PBS-1% Triton X-100 buffer (without protease and phosphatase inhibitors). Proteins were eluted with 30 μl of 1× LDS sample buffer (25 mm Tris-HCl, pH 8.0, 1% LDS, 10% glycerol, and 0.0045% bromophenol blue) for 20 min at room temperature in a thermomixer.

Protein eluates (15 μl, equivalent to 50–100 μg of total input protein) were separated by SDS-PAGE on 7.5% Stain-free TGX precast gels (Bio-Rad) and transferred to PVDF membranes. Membranes were blocked with 5% Phospho blocker (Cell Biolabs, Inc. CA, Cat No. AKR-103) at room temperature for 1 h and then incubated with an anti-phospho-serine/threonine motif antibody against the motif (Y/F/W) (S∗/T∗) or (S∗/T∗)F (Cell Signaling Technology Cat. No. 9631) at 1:2000 dilution in TBST with 5% Phospho blocker to identify phosphorylated serine 823 in TRPV4. Blots were then washed with TBST before the addition of an HRP-conjugated mouse anti-rabbit IgG (conformation-specific) antibody (Cell Signaling Technology Cat. No. 5127S, clone L27A9) at 1:3000 dilution in TBST with 2% NFDM at room temperature for 1 h. Membranes were repeatedly washed and developed using the ECL femto reagent (Thermo Scientific). The same membrane was stripped in Restore Western blot stripping buffer (Bio-Rad) and re-probed with an anti-DDK/FLAG antibody (1:5000 in TBST with 5% NFDM) (cat no. 14793S, Cell Signaling Technology), followed by a mouse anti-rabbit antibody as above (1:10,000 in TBST with 5% NFDM) to obtain the total TRPV4 signal.

### Biotinylation of cell surface proteins

A cell surface protein isolation kit (Pierce) was used to isolate the plasma membrane proteins from HCAECs according to the manufacturer’s protocol (54). Cells were incubated in PBS with 0.5 mg/ml sulfosuccinimidyl 2-(biotiamido) ethyl-1.3-dithiopropionate (Sulfo-NHS-SS-Biotin) for 30 min at 4 °C for labeling the cell surface protein. The reaction was stopped by adding a quenching solution, and cells were washed with ice-cold Tris-buffered saline (TBS), followed by total protein preparation using the procedure described in the previous section. Protein samples (150–200 μg input protein) were mixed with 50 μl of Neutr Avidin agarose beads (supplied as 50% slurry) and rotated for 3 h at 4 °C to isolate the biotinylated proteins, followed by bead collection by centrifugation at 2500*g* for 2 min and five washes with wash buffer. Proteins were eluted with 100 μl of 1× Laemmli sample buffer (Bio-Rad) by heating the samples for 5 min at 95 °C. The eluted proteins were analyzed by western blotting.

### Mass spectrometric identification of phosphorylation sites in TRPV4

HCAECs and HEK 293 cells transfected with TRPV4-GFP were used for protein isolation in a lysis buffer containing 25 mM Tris-HCl, pH 7.5, 150 mM NaCl, 1.0% Nonidet P-40, 0.5% sodium deoxycholate, 1 mM EDTA, and 5% glycerol, supplemented with 10 μM cantharidin, 1X protease inhibitors (Roche) and 1X phosphatase inhibitors (Pierce). The protein samples (200–250 ug) were separated on the 7.5% TGX gel (Bio-Rad). The obtained gel was stained with Imperial protein stain (Thermo Scientific, # 24615) for 1 h with gentle shaking at room temperature, followed by several washes with ultra-pure water till the optimal bands were observed. The gel bands were then excised and analyzed for protein phosphorylation by the Taplin Mass Spectrometry Facility at Harvard University. Briefly, excised gel bands were reduced with 1 mM DTT for 30 min at 60 °C and then alkylated with 5 mM iodoacetamide for 15 min. Gel pieces were then subjected to a modified gel trypsin digestion as seen in previous studies (76). Subsequently, gel pieces were dehydrated with acetonitrile for 10 min and dried in a speed-vac followed by rehydration with 50 mM ammonium bicarbonate solution containing 12.5 ng/μl modified sequencing-grade trypsin (Promega, Madison, WI) at 4 °C. Samples were then placed in a 37 °C room overnight and peptides were extracted by removing the ammonium bicarbonate solution. The extracts were then dried in a speed-vac (∼1 h). Samples were reconstituted in 5 to 10 μl of HPLC solvent A (2.5% acetonitrile, 0.1% formic acid). A nano-scale reverse-phase HPLC capillary column was created by packing 2.6 μm C18 spherical silica beads into a fused silica capillary (100 μm inner diameter x ∼30 cm length) with a flame-drawn tip (77). After equilibrating the column, each sample was loaded *via* a Famos autosampler (LC Packings) onto the column. A gradient was formed, and peptides were eluted with increasing concentrations of solvent B (97.5% acetonitrile, 0.1% formic acid). Eluted peptides were subjected to electrospray ionization in an LTQ Orbitrap Velos Pro ion-trap mass spectrometer (Thermo Fisher Scientific). Eluting peptides were detected, isolated, and fragmented to produce a tandem mass spectrum of specific fragment ions for each peptide. Peptide sequences (and hence protein identity) were determined by matching protein or translated nucleotide databases with the acquired fragmentation pattern by the software program, Sequest (Thermo Finnigan) (78). The modification of 79.9663 mass units to serine, threonine, and tyrosine was included in the database searches to determine phosphopeptides. Phosphorylation assignments were determined by the Ascore algorithm (79). All databases included a reversed version of all the sequences and the data was filtered between a one and two percent peptide false discovery rate.

### Homology modeling and computational refinement of the structure

We previously proposed a homology model of the TRPV4 channel (54) and simulated it with the putative docked location of the C-terminal helix. However, that model was based on the consensus results from multiple prediction servers and was mostly dominated by the template of the TRPV2 structure (PDB ID 5HI9) as there were no TRPV4 channels resolved by the time of the model assembly. In this article, we refined the model based on the actual TRPV4 structure (PDB ID 6BBJ). We have used the SwissPDB server (80) to create a full-length model for human TRPV4 on this template and transformed our original model to fit the resolved parts of TRPV4 structure while allowing the rest of the model that was not resolved in 6BBJ (including the C-terminal domain) to follow the transformation passively. The transition was performed using the Targeted molecular Dynamics protocol in NAMD (81) over 5 ns the simulation was performed using the CHARMM36 force field (82). The all-atom system included an explicit lipid bilayer and was performed in an NPT ensemble, flexible simulation cell, periodic boundary conditions, with TIP3P water model (83), and PME electrostatics (84) with 12 Å cut-off, rigid hydrogen bonds, and 2 fs timestep. Lastly, the structural visualization was done using VMD (85). The transformed model was simulated with the backbone restrained in the new positions for 20 ns to allow membrane lipids adjustment to the new conformation, and then the model was refined through four cycles of intermittent 5 ns unrestrained simulation and 1 ns symmetry-driven annealing that was previously shown to improve the homology models of the membrane channels (86).

### Data and statistical analysis

Calcium imaging experiments are presented as mean ± SD, with the number of independent experiments (*n*) included in brackets above the error bars or the legend. Each experiment had ≥ 20 randomly chosen cells recorded and averaged as one measurement. For Western blot experiments shown in Figure 2, TRPV4 pS/T(F/Y/W) band densities were normalized to TRPV4 expression levels and then converted to fold changes compared with WT vehicle control. For biotinylation experiments shown in Figure 3, ratios of the cell surface to total levels of TRPV4 protein for S823A, S823A/S824A, and S823E mutants were compared with WT TRPV4 in three individual experiments. Statistical comparisons were made by Student’s *t* test, one-way ANOVA, or two-way ANOVA when appropriate, using the respective statistical analysis programs provided in Sigma Plot (version 15). Significance in figures was depicted as ∗ or #, *p* < 0.05; ∗∗ or ##, *p* < 0.01.

## Data availability

All data are contained in the manuscript and the associated supporting information.

## Supporting information

This article contains supporting information.

## Conflict of interest

The authors declare that they have no conflicts of interest with the contents of this article.
