# Supplementary material for: Phosphorylation of distal C-terminal residues promotes TRPV4 channel activation in response to arachidonic acid
Source: J Biol Chem. 2025 Feb 3;301(3):108260. doi: 10.1016/j.jbc.2025.108260 (PMC11903807; doi:10.1016/j.jbc.2025.108260)
Supplement: Supplemental information [file mmc1.pdf]

Supporting Information (Figures S1-S5)

Phosphorylation of distal C-terminal residues promotes TRPV4 channel activation in response to arachidonic acid

Aravind Parthasarathy, Andriy Anishkin, Yangjing Xie, Kostiantyn Drachuk,
Yoshinori Nishijma, Juan Fang, Sevasti B. Koukouritaki, David A. Wilcox, and David X. Zhang

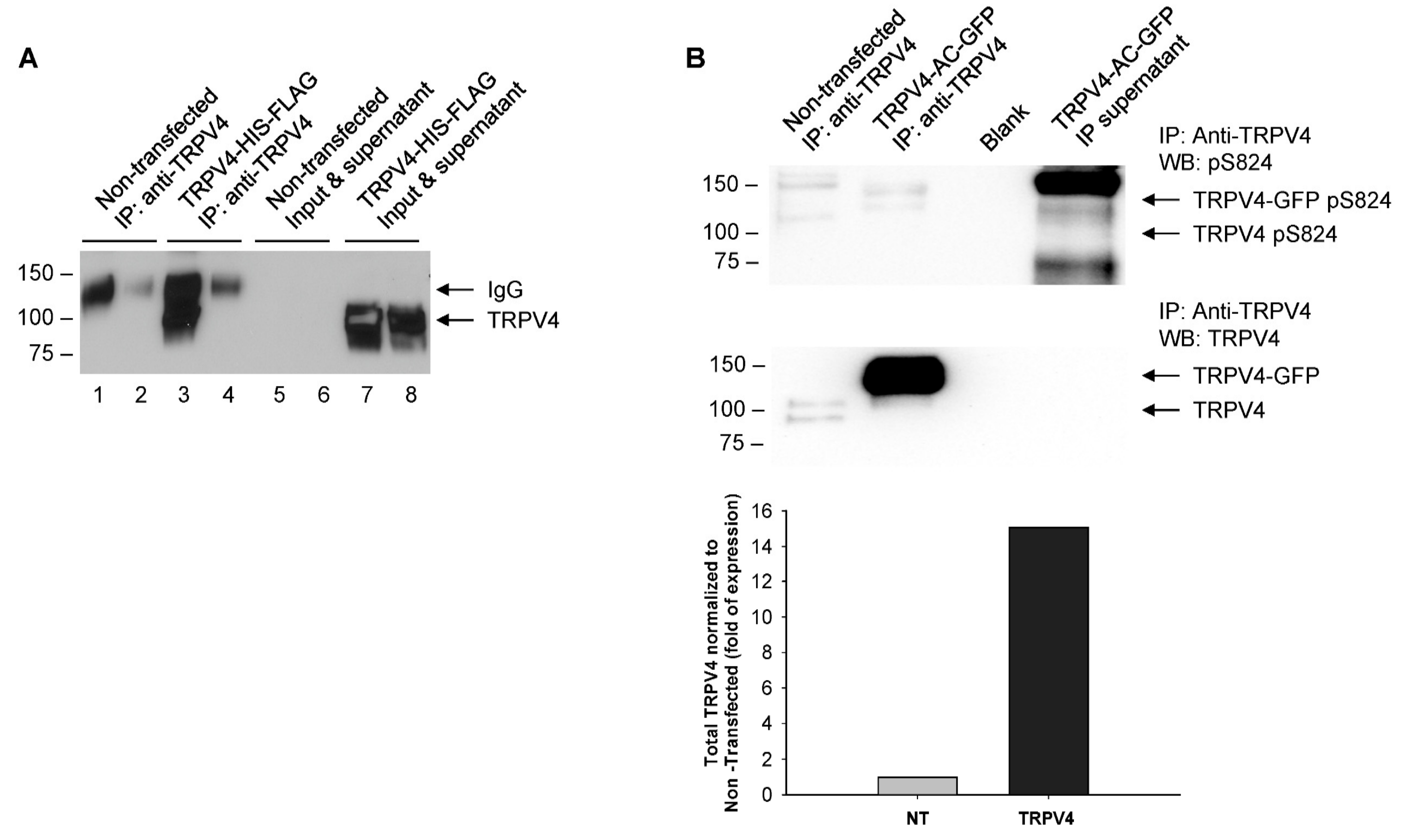

Fig. S1. A) Immunoprecipitation of TRPV4 from HEK293 cells with and without TRPV4-AN-HIS-DDK transfection. Lane 1: IP eluate 1 (25 µg input), lane 2: IP eluate 2, lane 3: IP eluate 1 (25 µg input), lane 4: IP eluate 2, lane 5: 25% input (5 µg), lane 6: IP supernatant (3.5 µg input), lane 7: 25% input (5 µg), lane 8: IP supernatant (3.5 µg input). Western blot analysis was performed using the same TRPV4 antibody (Cell Signaling #65893) as used for immunoprecipitation. A conformation-specific anti-rabbit IgG, HRP-linked Antibody (Cell Signaling #5127) was used as the secondary antibody. **B) Immunoprecipitation of TRPV4 from HCAEC with and without TRPV4-AC-GFP lentiviral transduction.** Lane 1: IP eluate of NT (150 µg input), lane 2: IP eluate of TRPV4-AC-GFP-overexpressing HCAEC (150 µg input), lane 3: Blank (Lysis Buffer), lane 4: IP supernatant of TRPV4-AC-GFP-overexpressing HCAEC (5 µg input). Western blot analysis was performed using a phosphoserine motif antibody against the motif RXRXXS*/T* (pSer-824 antibodies), and the same blot was re-probed with TRPV4 antibody (Cell Signaling #65893). A conformation-specific anti-rabbit IgG, HRP-linked Antibody (Cell Signaling #5127) was used as the secondary antibody. Quantification of endogenous and overexpressing TRPV4 in HCAEC is presented below the representative immunoblots shown in panel B.

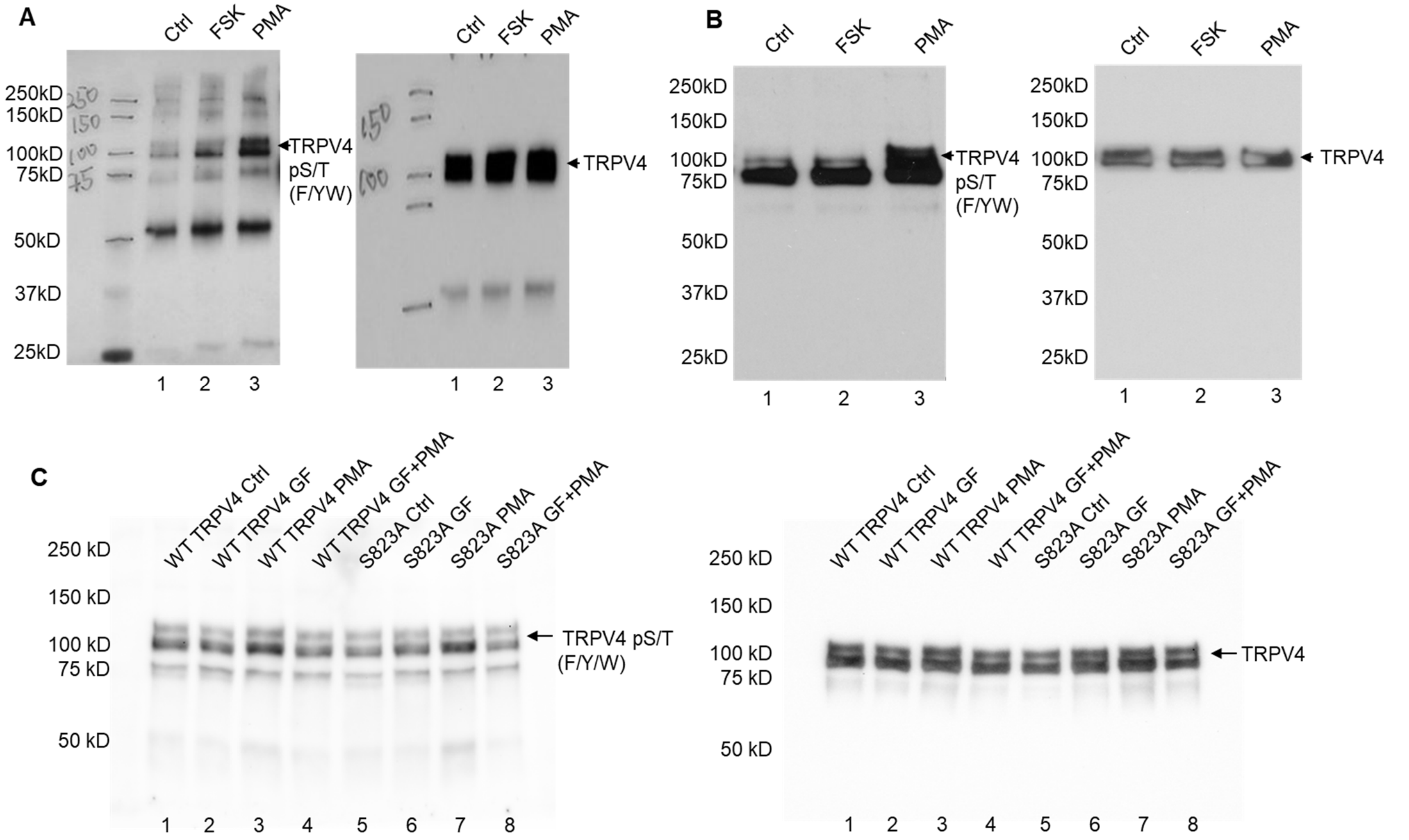

Fig. S2. TRPV4 phosphorylation in response to PKA and PKC activation in HCAECs and HEK 293 cells. Data represent uncropped blots of Fig 2 A-C. **A)** HCAECs were transduced with plasmids encoding TRPV4-HIS-FLAG in a lentiviral vector, and **B)** HEK 293 cells transfected with TRPV4-HIS-FLAG plasmids by lipofectamine. Lane 1: Control IP eluate of TRPV4 HIS-FLAG, lane 2: IP eluate of TRPV4 HIS-FLAG with FSK stimulation, lane 3: IP eluate of TRPV4 HIS-FLAG with PMA stimulation. After immunoprecipitation with anti-HIS or anti-FLAG antibodies, TRPV4 phosphorylation at Ser-823 or other unknown sites was analyzed by Western blotting using a phospho-(Ser/Thr) motif antibody that detects phospho-serine. For total TRPV4 detection, the same membrane was re-probed with anti-HIS antibodies for HCAEC or anti-FLAG antibodies for HEK 293 cells. **C)** HEK 293 cells transfected with TRPV4-HIS-FLAG WT or S823A plasmids were treated with PKC inhibitor GF 109203X (1 μM) for 30 mins, PKC agonist PMA (1 μM) for 30 mins, or a combination of GF 109203X and PMA for 30 mins each. Western blot analysis was performed as described in A-B.

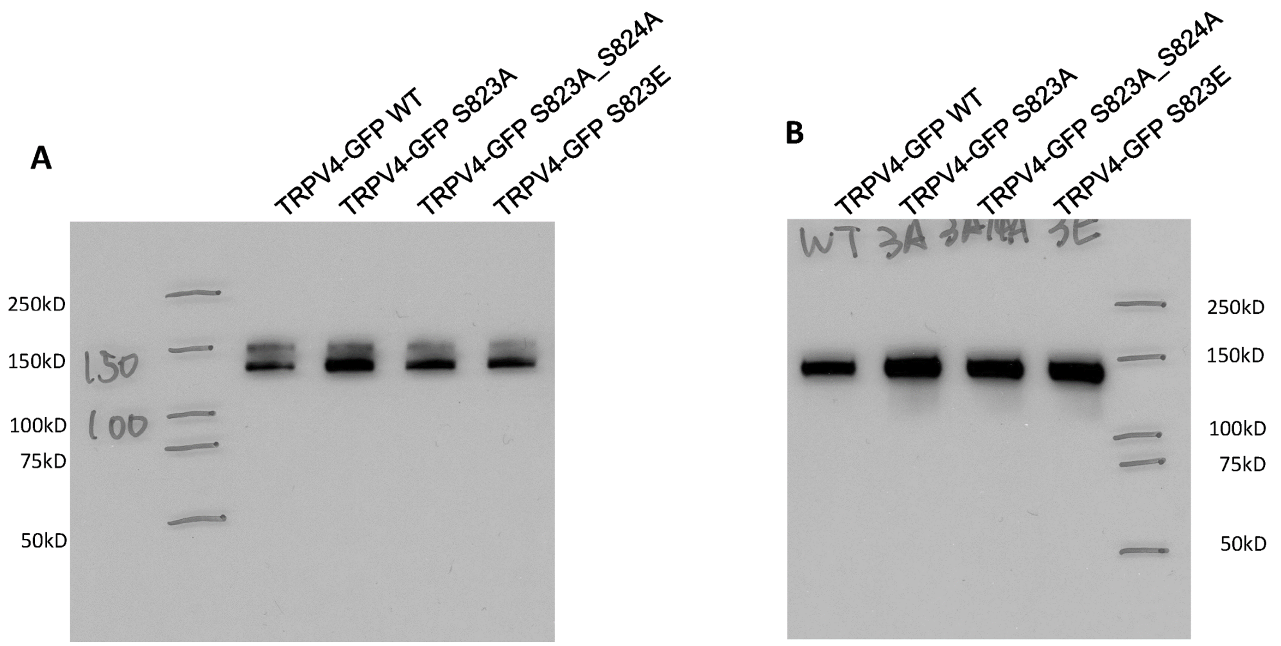

Fig. S3. TRPV4 containing S823A, S823A/S824A, or S823E substitution localizes on the cell surface of HCAECs. Data represent uncropped blots of Fig 5A. **A)** Cell surface proteins were labeled using a cell surface biotinylation method and captured with NeutrAvidin agarose beads. Protein eluates of HCAECs with TRPV4-GFP WT (lane 1), TRPV4-GFP S823A (lane 2), TRPV4-GFP S823A_S824A (lane 3), TRPV4-GFP S823E (lane 4)**. B)** Total cellular lysates were analyzed in parallel. The cell surface and total TRPV4 was detected using an antibody against GFP.

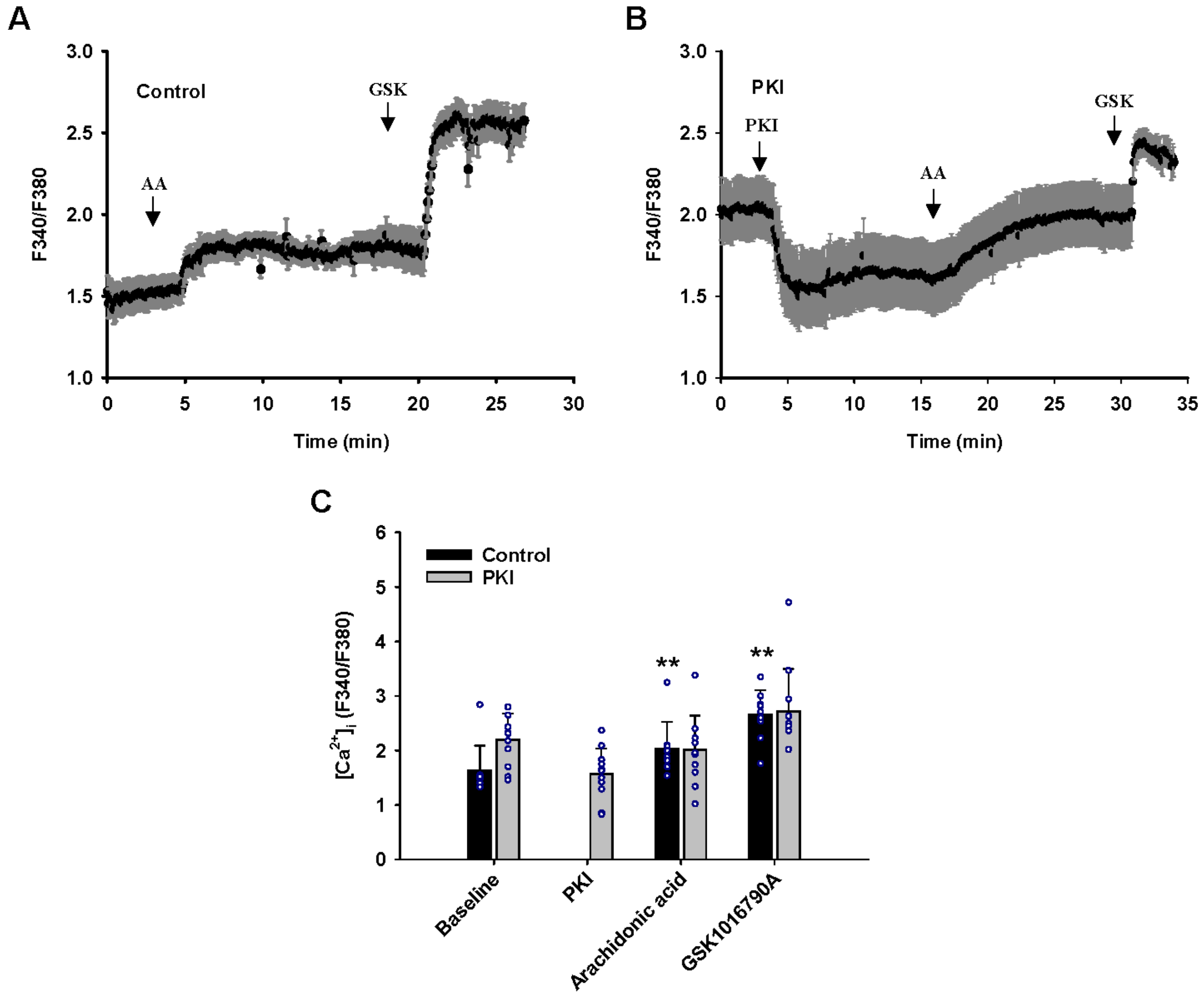

Figure S4. Inhibitory effect of PKI 14-22, a protein kinase A inhibitor, on arachidonic acid-induced TRPV4 activation in HEK 293 cells transiently expressing TRPV4-GFP and with baseline F340/F380 ratios >1.0. Cells were stimulated with arachidonic acid (AA; 6 μM), followed by synthetic agonist GSK1016790A (GSK; 10 nM) with and without pretreatment with PKI 14-22 (PKI; 1 μM). **A-B)** Representative traces of fura-2 calcium assay where the black line denotes mean F340/380 ratios and the error bars 1 × S.E. **C)** Summarized data for intracellular calcium concentrations ($[Ca^{2+}]_i$) in control (n=10) and PKI-treated (n=9) HEK 293 cells. All data represent mean ± S.D. $^{**}p < 0.01$ compared with baseline.

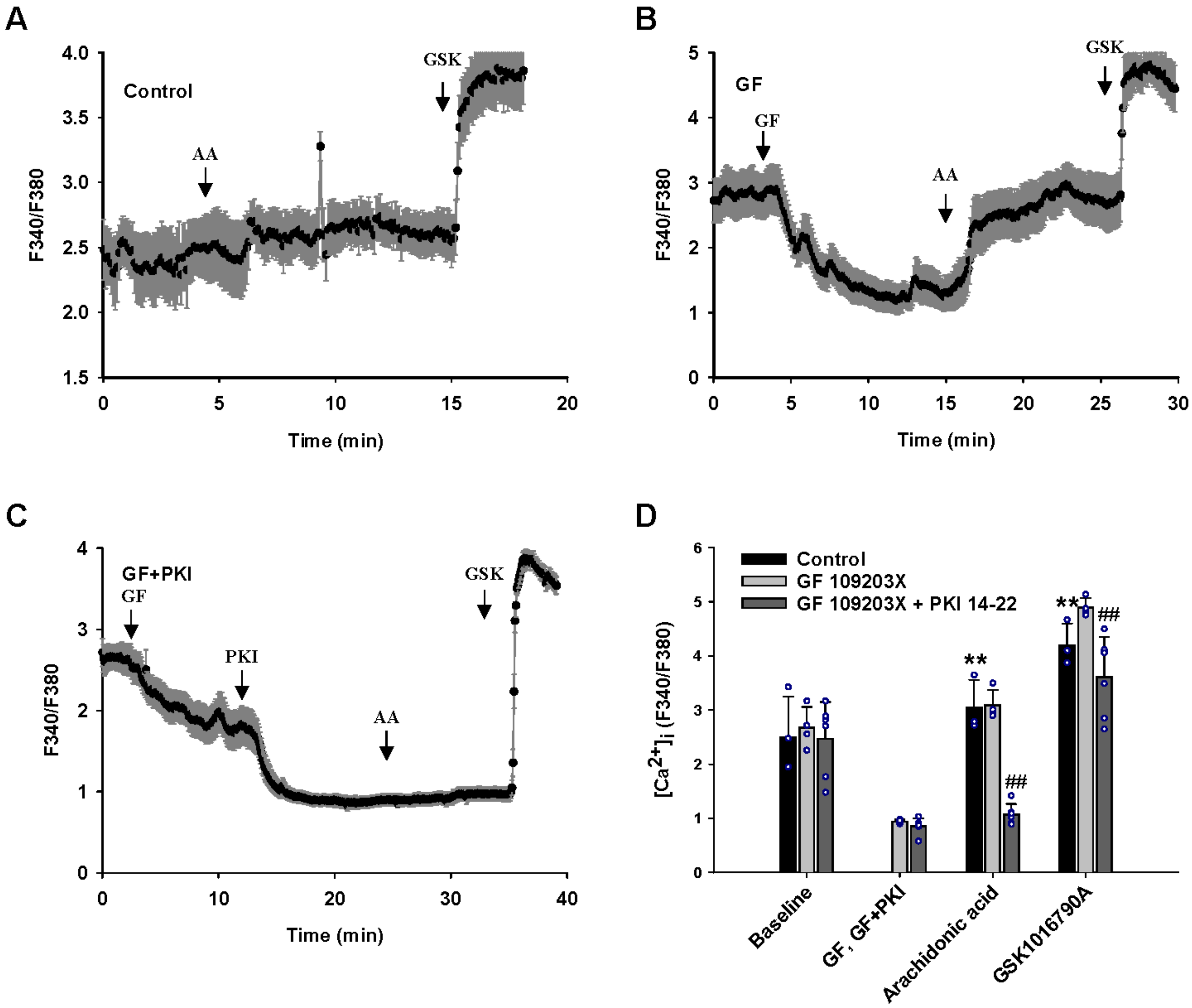

Figure S5. Effect of GF 109203X, a protein kinase C inhibitor, alone and in combination with PKI 14-22 on arachidonic acid-induced TRPV4 activation in HEK 293 cells transiently expressing TRPV4-GFP and with baseline F340/F380 ratios >1.0. Cells were stimulated with arachidonic acid (AA; 6 μM), followed by synthetic agonist GSK1016790A (GSK; 10 nM), with and without pretreatment with GF 109203X (GF, 1 μM) alone or in combination with PKI 14-22 (PKI; 1 μM). **A-C)** Representative traces of fura-2 calcium assay where the black line denotes mean F340/380 ratios and the error bars 1 × S.E. **D)** Summarized data for intracellular calcium concentrations ($[Ca^{2+}]_i$) in control (n=3), GF-treated (n=4), and GF+PKI-treated (n=6) HEK 293 cells. All data represent mean ± S.D. $^{**}P < 0.01$ compared with baseline, $^{\#\#}P<0.01$ vs compared with control.
